# Dataset on effect of sand grain size and water salinity on oil recovery

**DOI:** 10.1016/j.dib.2020.106695

**Published:** 2020-12-26

**Authors:** Emeka Emmanuel Okoro, Abdul-kabir Oluwaseyi Lawal, Samuel E. Sanni, Kale B. Orodu, Moses E. Emetere

**Affiliations:** a Petroleum Engineering Department, Covenant University Ota, Nigeria; b Chemical Engineering Department, Covenant University Ota, Nigeria; c Physics Department, Covenant University Ota, Nigeria

**Keywords:** Grain size, Oil recovery, Behavioral trend, Sandstone

## Abstract

This research investigates the combined effect of grain size and water salinity on oil recovery. Water flooding experiment was carried out using unconsolidated formation from Niger Delta. Five groups consisting of five samples, were tested for the effective interaction of two factors (grain size and salinity) and how they affect oil recovery. Each group was assigned a particular grain size while the prepared brine concentration was varied within a specified range. The selected grain sizes were obtained from laboratory sieve analyses. For each sand sample, the same concentration of brine used in saturating it was poured into the accumulator and connected to the flooding tube to displace a column of crude oil. The control valve was opened to cause oil displacement. The amount of brine used to displace the crude oil was ten times the pore volume and all the oil in each sample was recovered by the saturated brine solution. Laboratory investigations show that oil recovery was highest for brine concentration of 15,000 ppm; this was also the case in relation to oil recovery and sand grain sizes.

## Specifications Table

**Table:** 

Subject	Petroleum and Chemical Engineering
Specific subject area	Enhanced Oil Recovery
Type of data	Table, Graph and Figure
How data were acquired	Sand flooding experiments were carried out using unconsolidated formation from the Niger Delta region. Five groups consisting of five samples each were prepared to effectively study the interactions of both selected parameters (grain size and salinity) during oil recovery. Each group was assigned a particular grain size while the prepared brine concentration was varied within a specified range. Crude oil was poured into the tank / accumulator and connected to the cylindrical tube in which flooding was stimulated and streamed via the flow lines, the flow control valve and end sterns. The crude oil was left to displace the brine in the sand sample until only crude oil began coming out of the core. The volume of the displaced water in the funnel is equal to the volume of the crude oil in the sample sand; this is termed the hydrocarbon pore volume. The core samples were first saturated with crude oil before commencing the core flooding experiments.
Data format	Raw and Analyzed
Parameters for data collection	Sand grain sizes of 45 to 300 μm were used throughout this experiment. Prior to this experiment, a control experiment was conducted with non-saline water flowing through the set-up containing the different sand cores; this is so as to clearly perceive any effect of salt in crude oil/ brine in contact with the sand particles afterwards. It was observed that all recoverable crude oil in the sample had been made possible by the displacing fluid/brine, thus, the recovered oil in the cylinder was then recorded.
Description of data collection	Sand flooding experiments were carried out using unconsolidated formation from the Niger Delta region. Five groups consisting of five samples each were prepared to effectively study the interactions of both selected parameters (grain size and salinity) during oil recovery. Each group was assigned a particular grain size while the prepared brine concentration was varied within a specified range. Sand grain sizes of 45 to 300 micron were used throughout this experiment. Sand properties such as bulk and pore volumes, porosity as well as, wet and dry weight for the various sample groups were analyzed. The grain volume was determined from the dry sample weight and the grain density. The pore volume was determined by measuring the effective porosity.
Data source location	Niger-Delta, South-South Geopolitical Zone of Nigeria
Data accessibility	With the article
Related research article	E.E. Okoro, A.-K. Lawal, K.B. Orodu, S.E. Sanni, M.E. Emetere, Understanding the Behavioral Trends of the Effect of water salinity and Sand Size on Oil Recovery in Sandstone Reservoirs, South African Journal of Chemical Engineering, 35 (2021) 44-52. https://doi.org/10.1016/j.sajce.2020.11.005

## Value of the Data

• The dataset provides information on the crude oil recovery efficiency of different sand groups for sandstone reservoirs at different salinity levels.
• The dataset will help researchers, production and reservoir engineers improve their understanding of the effects of sand grain size and brine solution in determining the optimum oil recovery efficiency in a sandstone reservoir.
• The dataset is useful as it provides a reference point for determining the highest recovery when considering the effect of sand grain size and water salinity for enhanced oil recovery process.

## 1 Data Description

Tertiary (enhanced) oil recovery is that additional recovery over and above what could be recovered by primary and secondary recovery methods. Various methods of enhanced oil recovery (EOR) are essentially designed to recover oil, commonly described as residual oil, left in the reservoir after both primary and secondary recovery methods have been exploited within the most economical limits [1], [2], [3]. Water flooding is currently the most preferred recovery technique for most reservoirs because of higher sustained oil production rates, and the overall recoveries, that are obtained compared with the case without any form of water injection [4], [5], [6]. Water flooding is relatively cheap, especially for offshore fields because of the ready availability of seawater, although care has to be taken to ensure that the injected water does not result in unwanted, adverse reactions in the reservoir [7,8].

In this research work, the experimentation of the combined effects of grain size and water salinity on oil recovery was investigated. Water flooding experiments were carried out using an unconsolidated formation from the Niger Delta region of Nigeria.

Five groups of sand consisting of five samples each, were tested in order to determine their effects of the selected variables (grain size and salinity) on oil recovery. Each group was assigned a particular grain size while the prepared brine concentration was varied within a specified range. The selected grain sizes were obtained from laboratory sieve analysis. Grain sizes of 45-300 microns were used throughout this experiment.

Concentration values of 0 to 20,000 ppm salt in water were applied in determining the porosity of sand samples at all stages of the experiment. Sand sample properties were measured and evaluated by conventional techniques. Table 1, Table 2, Table 3, Table 4, Table 5 present a summary of the appraised sand properties for various sample groups.

**Table 1:** Appraised sand properties for group A.

Sand Sample	Pipe Length, L (cm)	Pipe Diameter, D (cm)	Net Dry Weight of Sand (g)	Net Wet Weight of sand (g)	Bulk Volume, BV (cc)	Pore Volume, PV (cc)	Porosity, φ (%)
A1	5.94	3.30	104.74	125.05	50.80	20.08	39.53
A2	6.09	3.30	107.11	129.04	52.10	21.39	41.06
A3	6.06	3.30	114.16	136.21	51.81	21.43	41.35
A4	6.14	3.30	110.50	132.13	52.47	20.96	39.94
A5	6.14	3.30	114.21	135.77	52.53	20.81	39.61

**Table 2:** Appraised sand properties for group B.

Sand Sample	Pipe Length, L (cm)	Pipe Diameter, D (cm)	Net Dry Weight of sand (g)	Net Wet Weight of sand (g)	Bulk Volume, BV (cc)	Pore Volume, PV (cc)	Porosity, φ (%)
B1	6.18	3.30	109.53	132.15	52.89	22.36	42.27
B2	6.22	3.30	109.24	133.40	53.16	23.57	44.33
B3	6.21	3.30	111.12	134.86	53.10	23.07	43.46
B4	6.12	3.30	112.92	136.98	52.36	23.30	44.50
B5	6.22	3.30	111.15	135.33	53.16	23.33	43.90

**Table 3:** Appraised sand properties for group C.

Sand Sample	Pipe Length, L (cm)	Pipe Diameter, D (cm)	Net Dry Weight of sand (g)	Net Wet Weight of sand (g)	Bulk Volume, BV (cc)	Pore Volume, PV (cc)	Porosity, φ (%)
C1	6.13	3.30	112.12	135.53	52.44	23.14	44.13
C2	6.16	3.30	107.33	130.71	52.66	22.80	43.30
C3	6.00	3.30	107.01	129.57	51.32	21.93	42.72
C4	5.81	3.30	105.79	128.18	49.72	21.69	43.63
C5	6.17	3.30	110.59	133.70	52.73	22.30	42.30

**Table 4:** Appraised sand properties for group D.

Sand Sample	Pipe Length, L (cm)	Pipe Diameter, D (cm)	Net Dry Weight of sand (g)	Net Wet Weight of sand (g)	Bulk Volume, BV (cc)	Pore Volume, PV (cc)	Porosity, φ (%)
D1	6.01	3.30	107.59	130.77	51.40	22.92	44.58
D2	6.21	3.30	107.81	131.46	53.11	23.07	43.43
D3	6.18	3.30	109.94	133.48	52.84	22.87	43.29
D4	6.16	3.30	108.38	131.27	52.66	22.18	42.11
D5	6.11	3.30	111.32	136.20	52.25	24.01	45.96

**Table 5:** Appraised sand properties for group E.

Sand Sample	Pipe Length, L (cm)	Pipe Diameter, D (cm)	Net Dry Weight of Sand (g)	Net Wet Weight of sand (g)	Bulk Volume, BV (cc)	Pore Volume, PV (cc)	Porosity, φ (%)
E1	6.08	3.30	91.58	115.33	51.99	23.48	45.17
E2	6.06	3.30	90.56	115.01	51.87	23.85	45.99
E3	6.24	3.30	93.03	118.09	53.35	24.36	45.66
E4	6.07	3.30	87.14	110.68	51.90	22.81	43.95
E5	5.98	3.30	86.82	110.35	51.13	22.71	44.42

The core flooding experiment was conducted using the five groups of sand samples, all data recorded during the experiments are tabulated in Table 6, Table 7, Table 8, Table 9, Table 10.

**Table 6:** Appraised flooding data for group A.

Sand Sample	Grain Size (µm)	Salinity (ppm)	Salinity (Molarity)	Crude Injected (ml)	*S_wc_ (%)	Crude Recovered (ml)	Recovery Factor (%)	⁎⁎S_or_ (%)
A1	300	0	0.00	17.0	15.4	12.5	73.5	26.5
A2	300	5,000	0.14	16.0	25.2	12.0	75.0	25.0
A3	300	10,000	0.28	16.0	25.2	12.5	78.1	21.9
A4	300	15,000	0.42	17.0	19.0	14.0	82.4	17.6
A5	300	20,000	0.56	16.5	20.7	13.5	81.8	18.2

⁎ Connate Water Saturation.

⁎⁎ Residual Oil Saturation.

**Table 7:** Appraised flooding data for group B.

Sand Sample	Grain Size (µm)	Salinity (ppm)	Salinity (Molarity)	Crude Injected (ml)	*S_wc_ (%)	Crude Recovered (ml)	Recovery Factor (%)	⁎⁎S_or_ (%)
B1	250	0	0.00	17.5	21.9	12.5	71.4	28.6
B2	250	5,000	0.14	16.0	32.2	11.5	71.9	28.1
B3	250	10,000	0.28	16.0	30.7	12.0	75.0	25.0
B4	250	15,000	0.42	18.5	20.6	15.0	81.1	18.9
B5	250	20,000	0.56	15.5	33.5	12.5	80.6	19.4

⁎ Connate Water Saturation.

⁎⁎ Residual Oil Saturation.

**Table 8:** Appraised core flooding data for group C.

Sand Sample	Grain Size (µm)	Salinity (ppm)	Salinity (Molarity)	Crude Injected (ml)	S_wc_ (%)	Crude Recovered (ml)	Recovery Factor (%)	S_or_ (%)
C1	100	0	0.00	16.0	30.7	11.5	71.8	28.2
C2	100	5,000	0.14	15.5	32.0	11.0	71.0	29.0
C3	100	10,000	0.28	18.5	15.5	13.5	73.0	27.0
C4	100	15,000	0.42	17.0	21.7	13.5	79.4	20.6
C5	100	20,000	0.56	16.0	28.3	12.5	78.1	21.9

*Connate Water Saturation.

**Residual Oil Saturation.

**Table 9:** Appraised core flooding data for group D.

Core Sample	Grain Size (µm)	Salinity (ppm)	Salinity (Molarity)	Crude Injected (ml)	*S_wc_ (%)	Crude Recovered (ml)	Recovery Factor (%)	⁎⁎S_or_ (%)
D1	80	0	0.00	17.0	25.8	12.0	70.6	29.4
D2	80	5,000	0.14	16.5	28.6	12.0	72.7	27.3
D3	80	10,000	0.28	16.0	30.1	12.0	75.0	25.0
D4	80	15,000	0.42	16.5	25.7	13.5	81.8	18.2
D5	80	20,000	0.56	17.5	27.1	14.0	80.0	20.0

⁎ Connate Water Saturation.

⁎⁎ Residual Oil Saturation.

**Table 10:** Appraised flooding data for group E.

Sand Sample	Grain Size (µm)	Salinity (ppm)	Salinity (Molarity)	Crude Injected (ml)	*S_wc_ (%)	Crude Recovered (ml)	Recovery Factor (%)	⁎⁎S_or_ (%)
E1	45	0	0.00	18.5	21.3	13.0	70.3	29.7
E2	45	5,000	0.14	15.5	35.1	11.0	71.0	29.0
E3	45	10,000	0.28	18.0	25.9	13.0	72.2	27.8
E4	45	15,000	0.42	18.0	21.1	14.0	77.7	22.3
E5	45	20,000	0.56	16.5	27.3	13.0	78.8	21.2

⁎ Connate Water Saturation.

⁎⁎ Residual Oil Saturation.

**Fig. 1:**
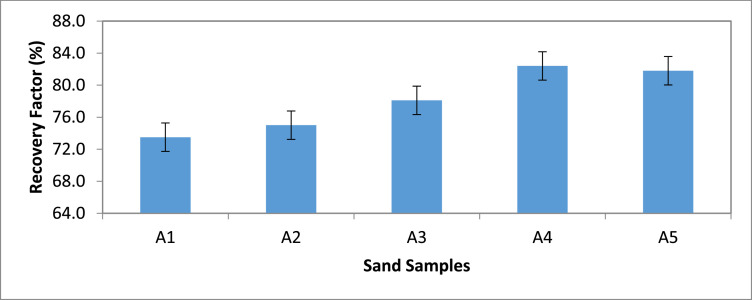
Recovery Factor for Group ‘A’ Samples.

**Fig. 2:**
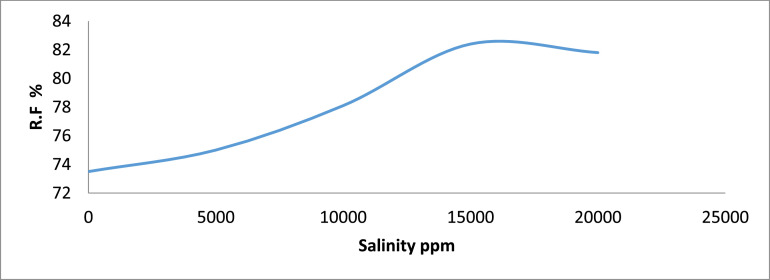
Recovery factor vs. salinity for Group A.

Category A: In this category, Sand sample A1 gave the least oil (i.e. 73.5%) when flooded with non-saline water (see Figs. 1 & 2). Sample A4 gave the highest oil recovery and the least residual oil saturation (17.6%).

The reservoir porosity is one of the important variables for EOR operations and low porosity will limit the applicability of the EOR methods. Among the existing hypotheses in enhanced oil recovery, wettability alteration towards increased water wetness is the widely suggested case of increased oil recovery. Literature have also shown through experimental analysis that changes in the injected brine composition can improve water flood performance [9].

**Fig. 3:**
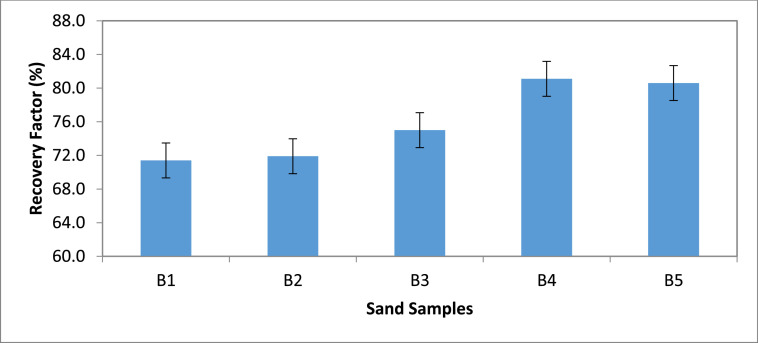
Recovery Factor for Group ‘B’ Samples.

**Fig. 4:**
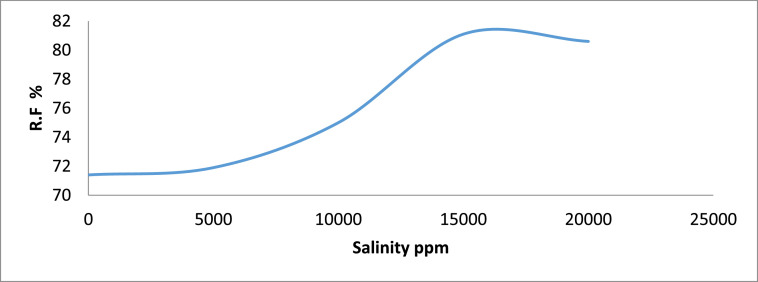
Recovery Factor vs salinity for group B.

Category B: The results show that sand sample B4 flooded with 15,000 ppm yielded the highest oil recovery of 81.1%, while sand sample B1 had the least oil recovery (71.4%) after being flooded with non-saline water (Figs. 3 & 4). Also, despite having the highest oil recovery, sample B4 gave the least residual oil saturation of 18.9%.

**Fig. 5:**
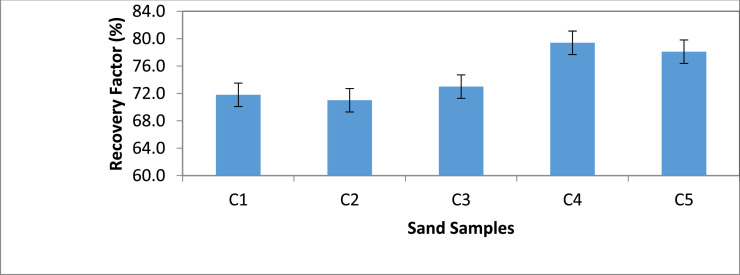
Recovery Factor for Group ‘C’ Samples.

**Fig. 6:**
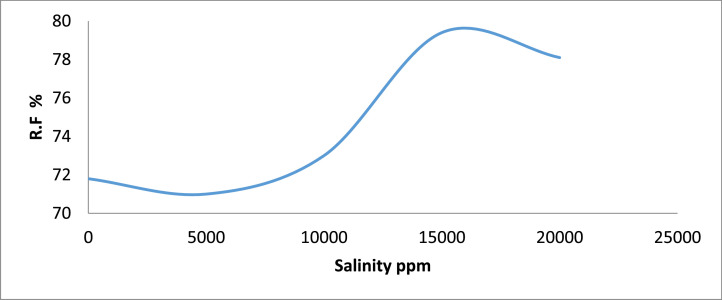
Recovery Factor vs salinity for group C.

Category C: In this category, Sand sample C2 recorded the least oil recovery of 71.8% when flooded with 5,000 ppm saline water (Figs. 5 & 6). It was observed that sample C4 with the highest oil recovery resulted in the least residual oil saturation of 20.6%.

**Fig. 7:**
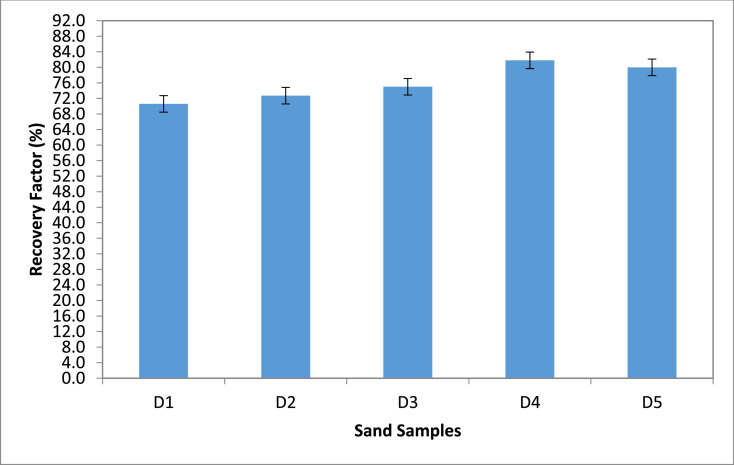
Recovery Factor for Group ‘D’ Samples.

**Fig. 8:**
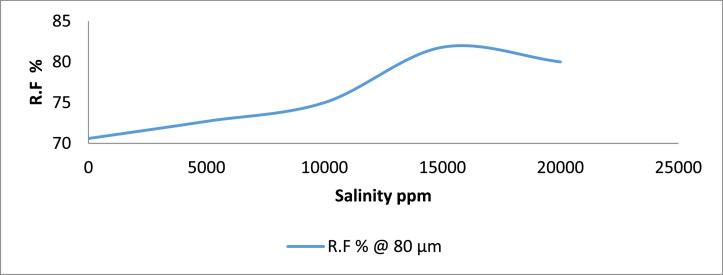
Recovery Factor vs salinity for group D.

Category D: Here, the results show that sand sample D4 that was flooded with 15,000 ppm gave an oil recovery of 81.8%. Sand sample D1 was seen to give the lowest oil recovery (70.6%) when in contact with no saline water (Figs. 7 & 8). Although, Sample D4 had the highest oil recovery, it was also seen to have the least residual oil saturation (18.2%).

**Fig. 9:**
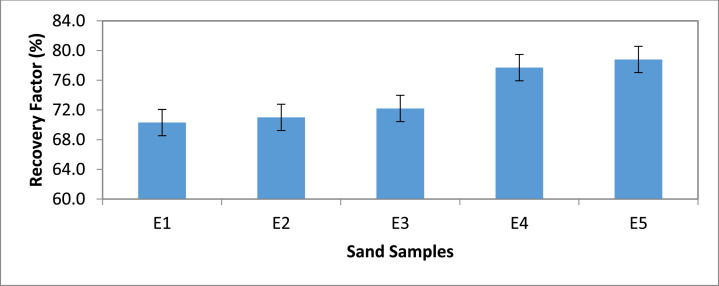
Recovery Factor for Group ‘E’ Samples.

**Fig. 10:**
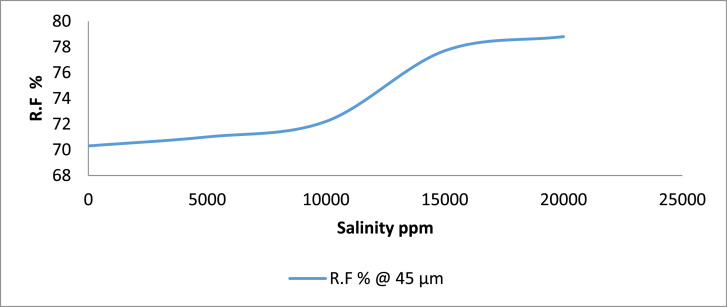
Recovery Factor vs salinity for group E.

Category E: Here, the results indicate that sample E5 which was contacted with 20,000 ppm gave the highest (78.8%) amount of oil. Sand sample E1 recorded the least oil recovery of 70.3% when flooded with no saline water (Figs. 9 & 10).

Table 11 shows the calculated recovery factor for salt concentrations of 5000 ppm, 10000 ppm, 15000 ppm and 20000 ppm for the varying sand grain sizes represented in Fig. 1 through to 10.

**Table 11:** Recovery factor for the different sand grain sizes.

Group A (300 µm)	Recovery Factor (%)	Group B (250 µm)	Recovery Factor (%)	Group C (100 µm)	Recovery Factor (%)	Group D (80 µm)	Recovery Factor (%)	Group E (45 µm)	Recovery Factor (%)
A1	73.5	B1	71.4	C1	71.8	D1	70.6	E1	70.3
A2	75.0	B2	71.9	C2	71.0	D2	72.7	E2	71.0
A3	78.1	B3	75.0	C3	73.0	D3	75.0	E3	72.2
A4	82.4	B4	81.1	C4	79.4	D4	81.8	E4	77.7
A5	81.8	B5	80.6	C5	78.1	D5	80.0	E5	78.8

During depletion, oil flows through the production wells to the surface because the pressure at the base of the well exceeds that exerted by the hydrostatic head of the column of oil in the well. Initially, this occurs naturally but over time the oil rate tends to decrease as the reservoir pressure decreases. In the absence of water injection, pumping may be used to maintain oil production rate at economic levels. If reservoir pressure falls below the oil bubble point pressure, the gas that was initially dissolved in the oil will come out of solution and, because it has a much lower viscosity, it will flow preferentially to the production well. At the same time the viscosity of the remaining oil increases, thus reducing its mobility further. This will in turn reduce the oil production rate [10]. Brine flooding is a recovery technique for most reservoirs because of the higher sustained oil production rates, and the overall higher recovery factor. The dataset can be used to propose ways of extending global oil reserves of sandstone reservoirs once oil prices are high enough to make these techniques economical. The realization of new giant fields is becoming increasingly difficult to find, thus, creating an avenue for extensive deployment of enhanced oil recovery. These datasets will help majority of oil companies producing from sandstone reservoirs to maximize their recovery factor from the oilfields as well as maintain an economic oil production rate.

## 2 Experimental Design, Materials and Methods

Sand flooding experiments were conducted in an unconsolidated formation. 5000-20,000 ppm brine solutions were prepared by dissolving 5, 10, 15 and 20 g of salt (laboratory NaCl) in 1000 mL of distilled water at ambient condition. The solution was stirred gently for about 30 minutes and left to stand for 24 hours for complete salt dissolution. The experimental set-up comprises of for this accumulators for holding the fluids (brine or crude oil), a cylindrical flooding tube, flow lines with control valves, and a separator (separating funnel); oil displacement was largely by gravitational pull.

Five groups of sand cores (A-E with sizes ranging from 45-300 microns) were selected to test for the influence of grain size and salinity on the amount of oil recovered. The saltwater/brine concentration was varied from 5000-20,000 ppm.

### 2.1 Determination of density

Density of brine was determined by the use of a density bottle. The empty bottle was weighed and its weight was recoread, and then it was filled with the desired fluid and also weighed; its new weight was recorded. Since the volume of the density bottle was known, the desired fluid densities were determined.

Weight of empty and dry bottle = m_1_

Weight of bottle with fluid = m_2_

Weight of fluid = m_2_-m_1_= m

Volume of fluid = 50ml

(1) $$\rho =\frac{m}{v}$$

where: v = volume of bottle is 50 mL

*ρ* = density of measured fluid, g/cm^3^

### 2.2 Determination of formation-sand porosity

The weight of each empty sample pack was measured and recorded and the weight of the sealed sample pack with the dry sand was also measured and recorded. Sample packs were saturated with brine in the desiccator in order to maintain uniformity and left for 48 hours. This was done for each of the prepared brine samples. The samples were brought out after 48 hours, after which they were wiped-clean and weighed again.

Pore Volume,

(2) $$\text{PV}=\frac{M_{\text{wet}}-M_{\text{dry}}}{\rho_{\text{brine}}}$$

where:

PV = pore volume of core sample, cm^3^

$M_{\text{wet}}$ = weight of wet core after saturation with brine, g

$M_{\text{dry}}$ = weight of dry core, g

$\rho_{\text{brine}}$ = density of brine, g/cm^3^

For estimation of Bulk Volume (BV), Eq. (3) was used.

(3) $$\text{BV}=\pi r^{2\;}\times \;h$$

where,

BV = bulk volume of core sample pack, cm^3^ h = length of core sample pack, cm r = radius of core sample pack, cm

Estimation of porosity,

(4) $$\Phi =\frac{\text{PV}}{\text{BV}}\times \;100\%$$

where:

Φ = porosity of core sample

### 2.3 Core-flooding experiments using crude oil

Crude oil was poured into the tank / accumulator and connected to flooding tube via the flow lines, flow control valve and the end sterns. The crude oil was then left to displace the brine in each sand sample until only crude oil began coming out of the core.

The volume of the displaced water in the funnel is equal to the volume of the crude oil in the sample sand. This is the hydrocarbon pore volume.

Determination of Connate Water Saturation

(5) $$S_{\text{wc}}=\frac{V_{\text{wc}}}{\text{HCPV}+V_{\text{wc}}}$$

where:

$S_{\text{wc}}$ = connate water saturation

$V_{\text{wc}}$ = connate water volume, cm^3^

HCPV = hydrocarbon pore volume, cm^3^

### 2.4 Core-flooding experiments with brine

For each sand sample, the same concentration of brine used in Section 2.2 was poured into the accumulator after which it was connected to the flooding tube to displace the crude oil. The control valve was opened and the quantity of brine used to displace the crude oil was observed to be ten times that of the sand sample having recovered all the oil. The recovered oil in the cylinder was then recorded.

Note: Recovered oil (N_P_) = oil displaced by the brine= volume of oil in the cylinder

Estimation of Recovery Efficiency:

(6) $$\%\;\text{Recovery}\;\text{Efficiency}=\frac{N_{p}}{\text{HCPV}}\times \;100\%$$

where:

$N_{p}$ = cumulative oil produced, cm^3^

## Ethics Statement

Not applicable.

## CRediT Author Statement

**Emeka Emmanuel Okoro:** Conceptualization; Methodology; Validation; Formal analysis; Writing - original draft; Supervision; **Abdul-kabir Oluwaseyi Lawal:** Conceptualization; Methodology; Investigation; Writing - original draft; **Samuel E. Sanni:** Methodology; Formal analysis; Writing - original draft; Writing - review & editing; **Kale B. Orodu:** Validation; Investigation **Moses E. Emetere:** Validation; Formal analysis.

## Declaration of Competing Interest

The authors declare that they have no known competing financial interests or personal relationships which have, or could be perceived to have, influenced the work reported in this article.
